# A novel registration method for long-serial section images of EM with a serial split technique based on unsupervised optical flow network

**DOI:** 10.1093/bioinformatics/btad436

**Published:** 2023-07-18

**Authors:** Tong Xin, Yanan Lv, Haoran Chen, Linlin Li, Lijun Shen, Guangcun Shan, Xi Chen, Hua Han

**Affiliations:** School of Artificial Intelligence, University of Chinese Academy of Sciences, Beijing 100190, China; State Key Laboratory of Multimodal Artificial Intelligence Systems, Institute of Automation, Chinese Academy of Sciences, Beijing 100190,China; School of Artificial Intelligence, University of Chinese Academy of Sciences, Beijing 100190, China; Institute of Automation, Chinese Academy of Sciences, Beijing 100190, China; School of Artificial Intelligence, University of Chinese Academy of Sciences, Beijing 100190, China; State Key Laboratory of Multimodal Artificial Intelligence Systems, Institute of Automation, Chinese Academy of Sciences, Beijing 100190,China; Institute of Automation, Chinese Academy of Sciences, Beijing 100190, China; Institute of Automation, Chinese Academy of Sciences, Beijing 100190, China; School of Instrumentation Science and Opto-electronics Engineering & Beijing Advanced Innovation Center for Big Data-based Precision Medicine, Beihang University, Beijing 100083, China; Institute of Automation, Chinese Academy of Sciences, Beijing 100190, China; State Key Laboratory of Multimodal Artificial Intelligence Systems, Institute of Automation, Chinese Academy of Sciences, Beijing 100190,China; School of Future Technology, University of Chinese Academy of Sciences, Beijing 100190, China

## Abstract

**Motivation:**

The registration of serial section electron microscope images is a critical step in reconstructing biological tissue volumes, and it aims to eliminate complex nonlinear deformations from sectioning and replicate the correct neurite structure. However, due to the inherent properties of biological structures and the challenges posed by section preparation of biological tissues, achieving an accurate registration of serial sections remains a significant challenge. Conventional nonlinear registration techniques, which are effective in eliminating nonlinear deformation, can also eliminate the natural morphological variation of neurites across sections. Additionally, accumulation of registration errors alters the neurite structure.

**Results:**

This article proposes a novel method for serial section registration that utilizes an unsupervised optical flow network to measure feature similarity rather than pixel similarity to eliminate nonlinear deformation and achieve pairwise registration between sections. The optical flow network is then employed to estimate and compensate for cumulative registration error, thereby allowing for the reconstruction of the structure of biological tissues. Based on the novel serial section registration method, a serial split technique is proposed for long-serial sections. Experimental results demonstrate that the state-of-the-art method proposed here effectively improves the spatial continuity of serial sections, leading to more accurate registration and improved reconstruction of the structure of biological tissues.

**Availability and implementation:**

The source code and data are available at https://github.com/TongXin-CASIA/EFSR.

## 1 Introduction

Serial section electron microscopy (ssEM) is a mainstream image-acquisition method for connectomics research. In ssEM, a biological tissue is cut into 30–50 nm thick sections and then imaged with a high-resolution electron microscope (EM) (Briggman and Bock 2012). This technique offers several advantages over serial block face scanning electron microscopy (Denk and Horstmann, 2004) and focused ion beam scanning electron microscopy (Knott et al., 2008), such as a large imaging area, parallel imaging in multiple EMs, and nondestructive sample processing, making it well suited to large-volume reconstruction. In recent years, ssEM has been utilized in many large-scale connectomics reconstruction projects (Hildebrand *et al.* 2017, Zheng *et al.* 2018, Macrina *et al.* 2021, Shapson-Coe *et al.* 2021).

One major drawback of ssEM is the physical cutting of the tissue block into sections, which results in the loss of continuity between sections. In addition, the section cutting process also introduces nonlinear deformations (Gardella *et al.* 2003). The destruction of biological structures caused by the loss of continuity and nonlinear deformations hinders the accurate reconstruction of these structures. To address these issues, serial section images need to be registered to restore the axial continuity of biological structures, such as neurites (Anderson *et al.* 2009, Cardona *et al.* 2010, Bock *et al.* 2011).

**Figure 1.**
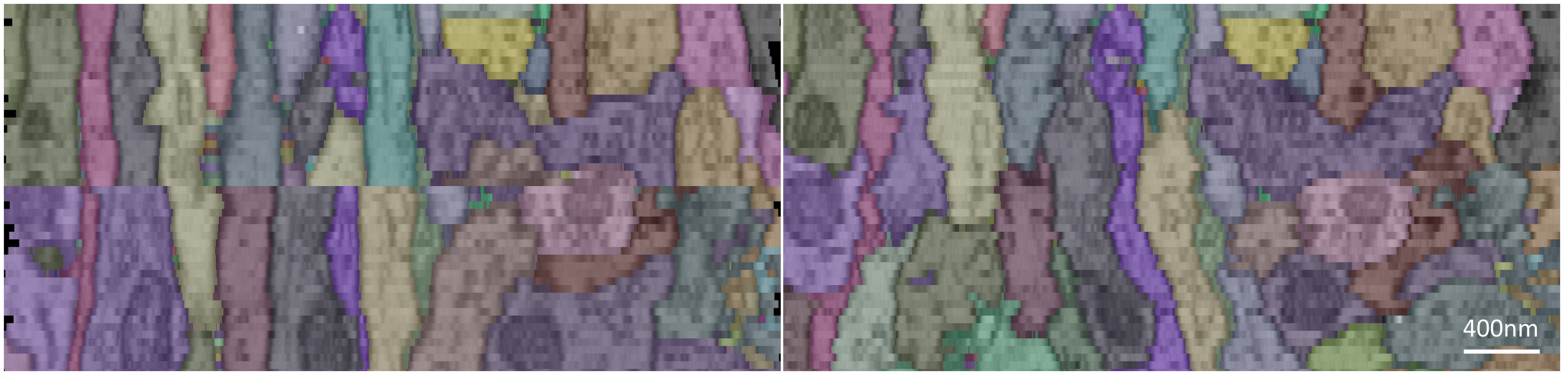
Longitudinal view of overregistration and error accumulation leading to structural breakage [left—result of ARFlow (Liu *et al.* 2020)] and that of ground truth (right) (different colors represent different neurites). The images are from CREMI dataset (https://cremi.org/).

Section registration involves aligning similar structures between adjacent sections to restore structure continuity in biological tissue. However, this task is challenging due to the similarity in morphology of different neurites, natural morphological changes of the same neurite across different sections, and factors, such as dust, radiation damage, and other artifacts. Several methods have been proposed for section registration, such as Elastic registration (Saalfeld *et al.* 2012), ssEMNet (Yoo *et al.* 2017), voxelMorph (Balakrishnan *et al.* 2019), and SEAMLeSS (Mitchell *et al.* 2019). However, these methods that measure pixel-level similarity between sections to perform registration can lead to overregistration, causing destruction of the axial structure of the biological tissue by incorrectly eliminating natural morphological changes between sections. As shown in Fig. 1, the strong similarity constraint leads to a reconstruction where all registered neurites are perpendicular to the section plane.

This study proposes a solution to tackle the issue of overregistration in biological tissue section registration by utilizing a feature representation that considers the morphological variations among sections to measure inter-section similarity. Convolutional neural networks are well suited to this task due to their superior nonlinear modeling capabilities and ability to learn feature representations from training data (Mishchuk *et al.* 2017, Tian *et al.* 2017). In particular, the Unsupervised Learning of Thickness-Insensitive Representations (UTR) (Xin *et al.* 2021) feature descriptor is employed as it accounts for the morphological variations of neurite structures and reduces the impact of biological morphological changes between sections on registration. This study uses the unsupervised optical flow network to register sections. By utilizing UTR descriptor to measure the similarity between sections during network training, the proposed method allows the optical flow network to focus on positional distortions rather than morphological changes in the neurite structure, thereby minimizing the risk of overregistration.

In addition to pairwise registration design concerns, avoiding errors cumulative during serial registration is also a significant challenge. Most existing registration algorithms follow a specific order, such as successively registering serial sections to the most recently registered sections from the top of the block (Tasdizen *et al.* 2010). However, this process introduces errors due to morphological changes in neurite structure, and the error can accumulate across sections. Severe error accumulation can cause the warped section to fail to meet the similarity constraint of the serial registration algorithm, leading to a break in the neurite structure, as shown in Fig. 1. Elastic registration (Saalfeld *et al.* 2012) constructs a spring mesh across the serial sections to avoid error accumulation. ssEMNet (Yoo *et al.* 2017) implements serial registration by incorporating supervision of neighboring sections. Popovych *et al.* (2020) proposed enhancing local continuity through vector voting with several neighboring sections. Lv *et al.* (2020) reduce error accumulation by imposing constraints on serial registration, but this method is restricted to rigid registration. To address error accumulation, this study proposes a structural regression method. The cumulative error is first estimated using optical flow, and then compensation is performed section by section. Since biological structures are expected to transform smoothly in the axial direction, the similarity between sections determines the degree of error compensation.

Although structural regression has the benefit of compensating for cumulative registration errors, it has limitations in that it can only be used in short series because the cylindrical model (see Section 2.2 for details) of neurites used in this article is only applicable to short series and may not be suitable for long series. Therefore, to enable the use of structural regression in long series, this article proposes a strategy of dividing the long series into multiple short series and performing registration on each of them separately. With this strategy, the proposed registration framework can be used for the registration of long series.

## 2 Materials and methods

**Figure 2.**
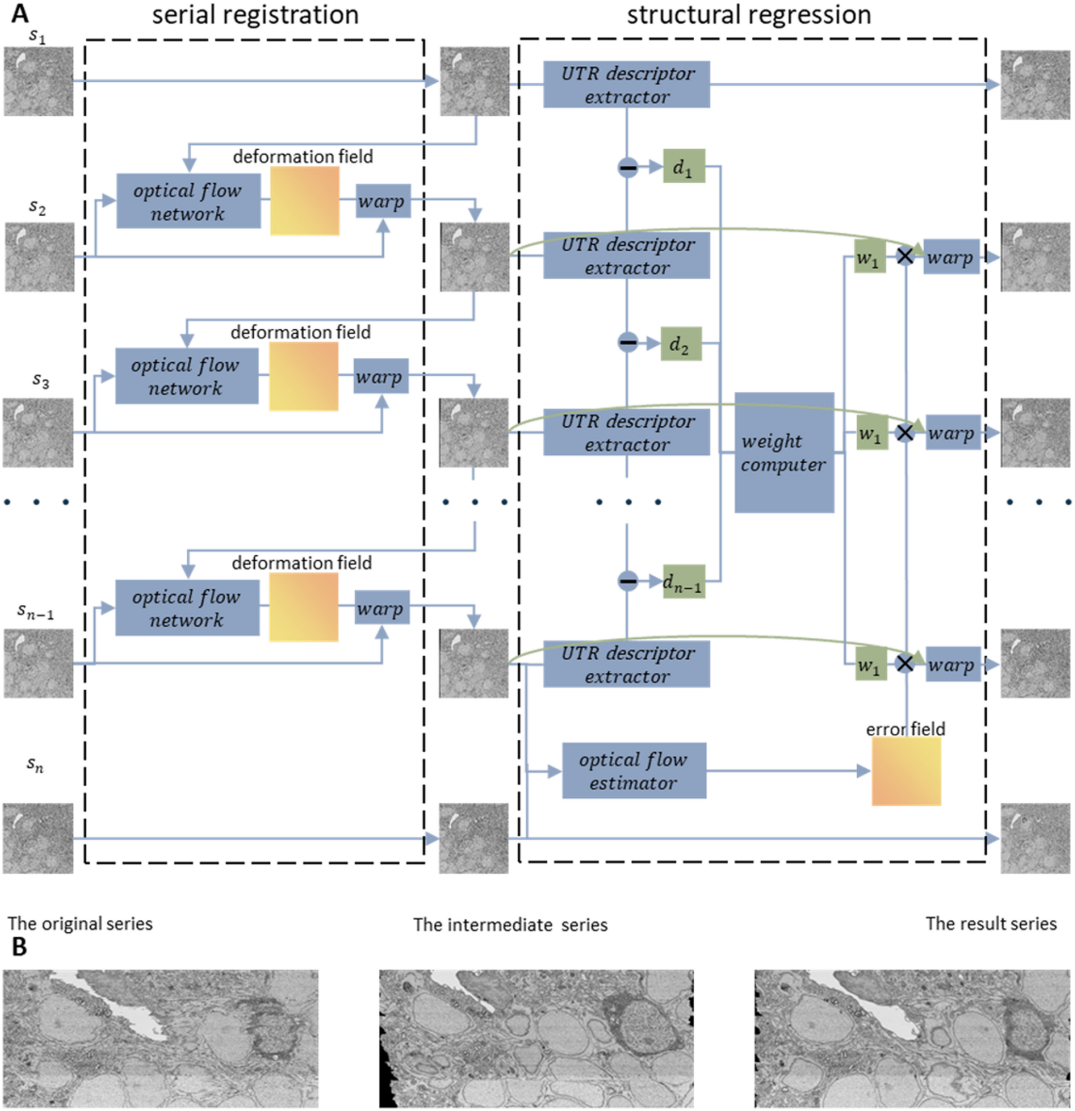
The overall serial registration pipeline and longitudinal view of the results for each step. (A) Serial sections are sequentially registered by the optical flow network and then structural regression is performed to generate the final results. (B) Longitudinal view diagram of the original, intermediate, and serial results. The series is composed of 15 short series for a large volume.

This study proposes a serial section registration framework (Fig. 2A) that aims to achieve accurate reconstruction of neurite structures. The framework is designed to address two main aspects of serial registration: preserving the natural morphology of neural structures and compensating for cumulative errors introduced during registration. To achieve these goals, the first and last sections are registered by rigid registration, with the purpose of restoring the positional relationship between the first and last sections to provide a reference for structural regression. The optical flow is then used to register the remaining sections sequentially. Cumulative error fields are estimated and eliminated by warping for structural regression. In this study, the original section i is denoted as $s_{i}$, and warped section i after registration is referred to as $ws_{i}$. The reference section is denoted as $s_{1}$, and the other sections are registered to the previous section sequentially. To register $s_{i},i=2,\ldots ,n-1$, the optical flow network E estimates the optical flow $F_{i\to i-1}=E\left(s_{i},ws_{i-1}\right)$ from $s_{i}$ to $ws_{i-1}$, and then $F_{i\to i-1}$ is used to warp $s_{i}$ to obtain $ws_{i}=\phi_{F}\left(s_{i}\right)$. The cumulative error is estimated as the optical flow from $ws_{n-1}$ to ${ws}_{n}$ and removed from ${ws}_{i},i=2,\ldots ,n-1$ after serial registration to obtain the final result.

### 2.1 Optical flow network

**Figure 3.**
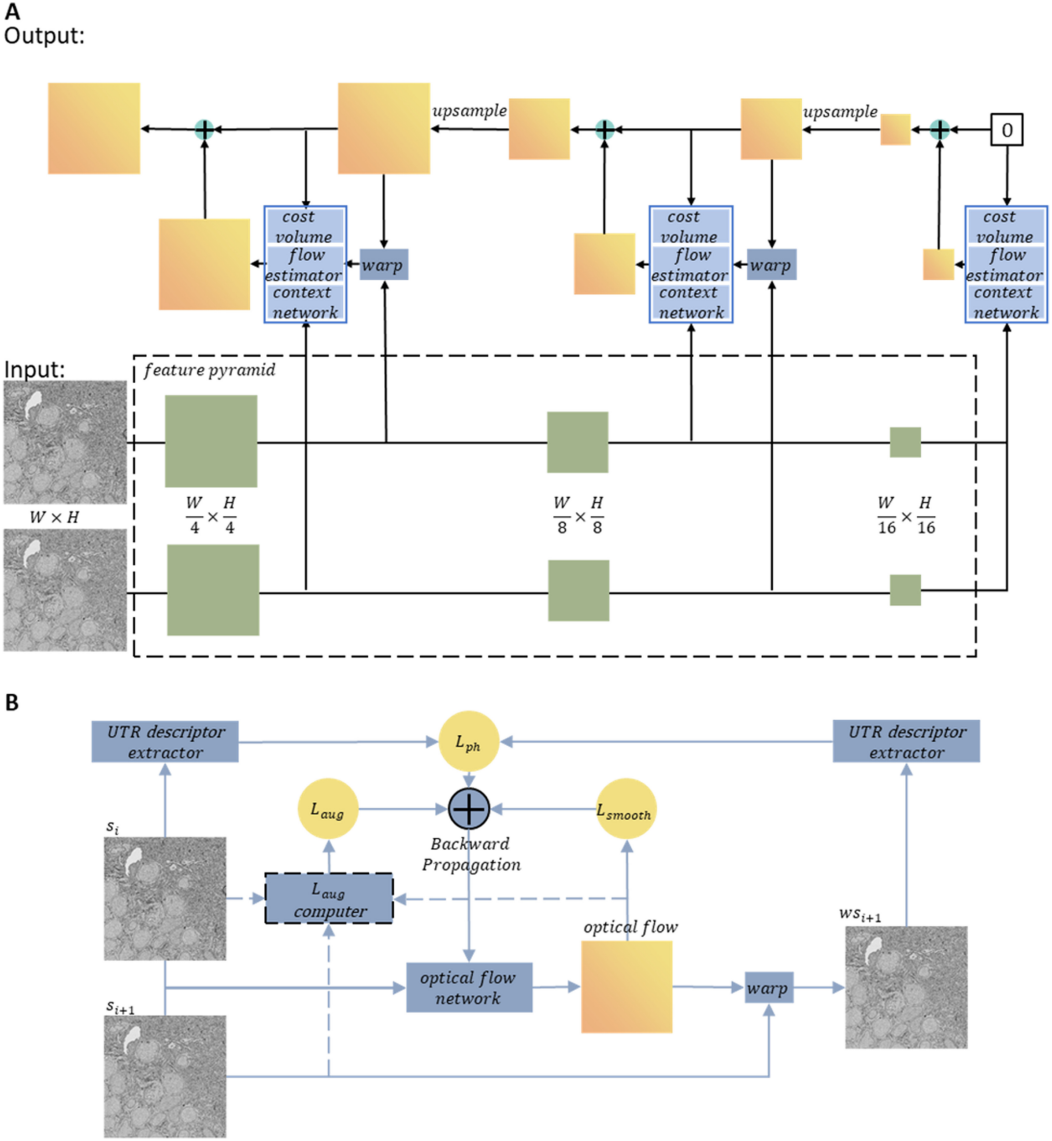
Architecture and training of the optical flow network. (A) The pwc-lite network architecture (the optical flow network used in this article has five octaves, only three of which are shown here for space limitations). (B) The training pipeline of the network, UTR descriptor is used to calculate $L_{\mathrm{ph}},\;L_{\mathrm{smooth}},\;L_{\mathrm{aug}}$ is calculated in the same way as ARFlow.

The serial registration process involves multiple pairwise registrations, so the choice of pairwise registration model can significantly impact the results. Common parametric models may not be sufficient to accurately model the complex nonlinear deformations introduced during section preparation. To address this challenge, this article uses an optical flow approach to register pairs of sections. For each pair of sections, this registration process involves estimating the optical flow field between the sections using an optical flow network and warping them into the reference section. This approach improves on the state-of-the-art unsupervised optical flow method ARFlow (Liu *et al.* 2020) to achieve better registration results for images of biological sections. Optical flow networks have a greater potential to learn the unique features of section images compared to traditional optical flow methods, and ARFlow uses unsupervised learning, avoiding the need for data labeling, which is difficult to obtain. The network architecture used in this article, identical to that of ARFlow, is a lightweight PWC-Net (Sun *et al.* 2018) (pwc-lite) that computes the optical flow from coarse to fine using five octaves; only three are shown in Fig. 3A due to space limitations.

The photometric loss $L_{\mathrm{ph}}$ in ARFlow measures the similarity between images and is a weighted sum of the L1 loss, structural similarity (ssim) loss, and ternary loss, which can only measure the pixel-level similarity between images. However, when this optical flow network is used to register images of biological sections, the differences in neurite structure morphology between adjacent sections are severely penalized, leading to the elimination of the morphological differences between sections of biological tissue during registration. To address this issue, the proposed method utilizes the pre-trained UTR (Xin *et al.* 2021) Network to extract descriptor features ($\mathrm{feat}\left(s_{i}\right)$) of sections $s_{i}$ to calculate the similarity between sections, thereby allowing the network to focus more on the location changes of neurite structure and minimize the impact of neurite structure morphology changes between sections on registration. Specifically, in this article, we define $L_{\mathrm{ph}}=\sqrt{{\left(\mathrm{feat}\left(s_{i-1}\right)-\mathrm{feat}\left(\phi_{F}\left(s_{i}\right)\right)\right)}^{2}\;}$, where $\phi_{F}\left(s_{i}\right)$ is the section image obtained by warping the section $s_{i}$ with the optical flow F. Additionally, the smoothness loss $L_{\mathrm{smooth}}$ constrains the consistency of the deformation field, while the augmentation loss $L_{\mathrm{aug}}$ augments data and improves the accuracy of unsupervised learning. Figure 3B illustrates the training process of the optical flow network, which involves optimizing the photometric loss $L_{\mathrm{ph}}$, the smoothness loss $L_{\mathrm{smooth}}$, and the augmentation loss $L_{\mathrm{aug}}$.

### 2.2 Structural regression

**Figure 4.**
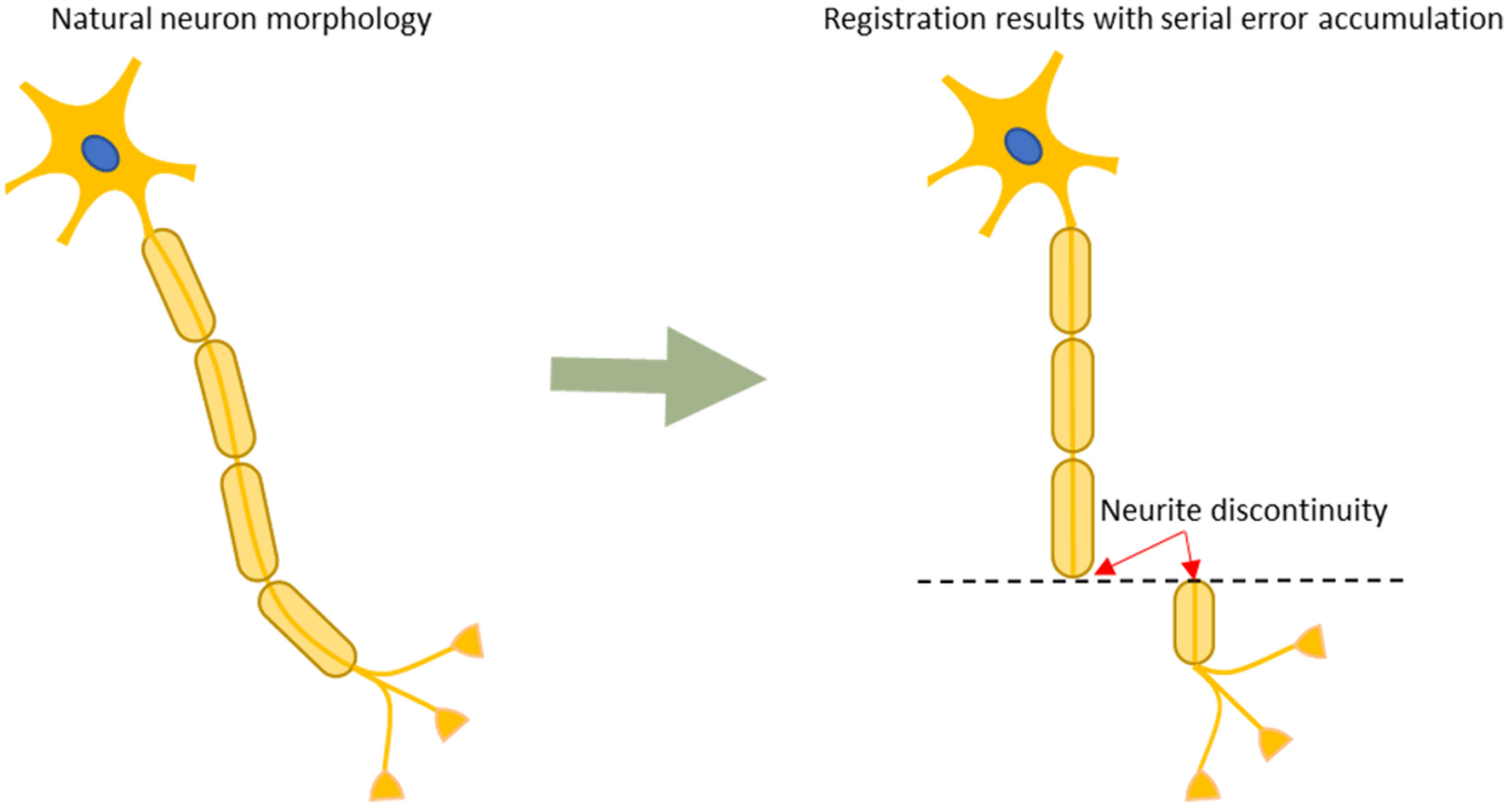
Accumulation of registration errors leads to distorted reconstruction results.

Most registration methods are used to register sections with a reference section, but they may warp the spatial structure of neurites and introduce errors. The sequential nature of the registration process can cause errors to accumulate, resulting in distorted neurites. As shown in Fig. 4, the cumulative errors tend to distort angled neurites to be perpendicular to the section plane. These errors can eventually become so significant that the matching algorithm cannot match adjacent sections, leading to a break in the neural structure’s continuity. Although the proposed improved optical flow method attempts to preserve the natural morphological variation of biological structure across sections, it still cannot completely prevent the accumulation of errors.

The neurite in a short series can be approximated as a cylinder, resulting in a consistent direction of the registration error across all sections. The cumulative error $e_{i}$ for a given section $s_{i}$ can be calculated as the sum of the registration errors up to that section, where $\Delta e_{i}$ represents the error on section $s_{i}$, such that $e_{i}=e_{i-1}+\Delta e_{i}=\sum_{j=1}^{i}\Delta e_{j}$.

To compensate for the cumulative registration error in each section, this study proposes using the UTR descriptors to estimate the proportion of the error on the current section. The difference in UTR descriptors between two adjacent sections is used to indicate the proportion of the cumulative error on the current section. To suppress the influence of cumulative errors and accurately reconstruct the morphological structure of neurites, the optical flow is used to warp section $s_{i}$ with a weight $w_{i}$ that is calculated based on the distances between the UTR descriptors of all sections.

Before the serial registration, the first and last sections have been already rigidly registered to recover their spatial position correlations, providing a reference for the subsequent structural regression. These sections are not deformed during the following registration process. The optical flow $F_{n-1\to n}$, which represents the displacement field of all pixels between the last two sections, can be used to approximate the total cumulative error $e_{n-1}$. The UTR descriptors prioritize the changes in position between sections, which makes them a more reasonable way to evaluate the degree of cumulative error.

To further improve the accuracy of the registration, this study uses the distances between UTR descriptors to calculate weights for all sections in the series. To calculate the weight $w_{i}$ for section $s_{i}$, the distance $d_{i}$ between the UTR descriptors of section $s_{i}$ and section $s_{i-1}$ is first calculated using the L2 norm, denoted by $d_{i}=\sqrt{{\left(\mathrm{feat}\left(s_{i}\right)-\mathrm{feat}\left(s_{i-1}\right)\right)}^{2}}$, where $\mathrm{feat}(s_{i})$ denotes the UTR descriptor of section $s_{i}$. The weight $w_{i}$ is then defined as the ratio of the sum of distances between the UTR descriptors of all sections up to section $s_{i}$ and the sum of distances between the UTR descriptors of all sections in the series, given by $w_{i}=\sum_{j=1}^{i}d_{j}/\sum_{j=1}^{n}d_{j}$. The weight $w_{i}$ does not directly measure the proportion of cumulative error on section $s_{i}$ relative to the total cumulative error on the series. However, it reflects the degree to which compensation is applied to the cumulative error on section $s_{i}$. The assumption that neurites are cylindrical in short series means that larger registration errors on a section correspond to greater differences between that section and the previous section. The difference in UTR descriptors reflects the size of the error on the current section after minimizing the impact of neurite structure changes, and therefore $w_{i}$ can reflect the degree of error compensation.

By using the weights to warp each section with the optical flow, this approach can effectively suppress the influence of the cumulative error and accurately reconstruct the morphological structure of neurites.

### 2.3 Division of long series

This article also proposes a method for registering long-serial sections by dividing them into multiple short series and registering them separately to compensate for the cumulative errors in serial registration of short series. Structural regression cannot accurately recover the structural morphology of neurites in longer series, as it is not possible to approximate the long neurites as cylinders. To address this issue, the long series is divided into n equal short series, where n is determined by the degree of error accumulation, with larger n used when the error accumulation is smaller and *vice versa*.

The last section of each short series, referred to as the benchmark section, also serves as the first section of the subsequent short series. The benchmark sections mentioned in this paragraph formed another series with lower axial resolution, and they are rigidly registered in advance by extracting SIFT points and matching them with RANSAC to estimate the rigid transformation matrix for registration. Those rigid transformations determine the spatial correspondence of the short series. This enables the recovery of position information for the first and last sections of each short series. Subsequently, the proposed short series registration method is applied to each short series for serial registration, and the resulting short series are stacked to obtain the registration results for the long series.

### 2.4 Implementation details and data description

This study utilizes three different datasets, namely the zebrafish brain ssEM data, CREMI dataset, and FIB-mito dataset. The zebrafish ssEM dataset, which is composed of 350 pairs of images for training and 100 pairs of images for validation, is utilized to train the optical flow network. The architecture of this network is identical to that of ARFlow. Unlike ARFlow, the photometric loss is based on UTR descriptors. To test the proposed method, the CREMI and FIB-mito datasets, which have different morphological appearance compared to the datasets used for training, are used as test sets. The optical flow network is tested on these test sets, and the results are evaluated.

The zebrafish ssEM dataset used in this study is obtained from the brain of a zebrafish larva using automatic tape-collecting ultra-microtome scanning electron microscope imaging, which was described in Xin *et al.* (2022). The dataset consists of 450 pairs randomly sampled brain sections with a voxel resolution of $32\times 32\;{\mathrm{nm}}^{2}$ and a section thickness of 30 nm. The image size is 1024×1024.

The CREMI dataset (https://cremi.org/) used in this study is acquired from the brain of an adult Drosophila using serial section transmission electron microscopy. The dataset consists of 125 successive EM images with a voxel resolution of $4\times 4\times 40\;{\mathrm{nm}}^{3}$ and an image size of 1250×1250. To simulate the real deformation during section preparation, the method of Yoo *et al.* (2017) is used to warp images. Thin plate spline (Bookstein 1989) is used to deform sections through multiple random vectors at random locations. The random vectors are sampled from a normal distribution with a mean of zero, and the random positions are distributed uniformly across the whole section image. In addition, these sections are centered-cropped to 1024×1024 for ease of use. The dataset is strictly registered manually and can be used as the ground truth for EM images.

The FIB-mito (Lucchi *et al.* 2013) dataset used in this study represents a $5\times 5\times 5\;µm^{3}\;$block taken from the CA1 hippocampus region of the brain. The dataset corresponds to a 1065×2048×1536 volume with a resolution of$\;5\times 5\times 5\;nm^{3}$, and it is acquired by FIB-SEM. To simulate the typical section thickness of ssEM, which is between 30 and 50 nm, one of every eight sections in this dataset is kept during the experiment (at a 40 nm interval). The test dataset is generated using the same method used for the CREMI dataset.

For the CREMI dataset, this study uses the normalized cross-correlation (NCC) and the Dice coefficient of neurite labels to evaluate the accuracy of the registration results. The Dice coefficient is a measure of set similarity. In registration, the Dice coefficient of the label indicates the overlap degree of the same neuron after registration, and the Dice coefficient tends to 1, indicating that the higher the accuracy of the recovery of the same neuron structure after registration is. That is, the higher the accuracy of the registration result is. Furthermore, the Dice coefficient can measure the registration accuracy not affected by the image quality. To prevent the impact of labels representing intercellular spaces, this study uses the Dice coefficient of 50 neurites with the largest area on the first section to measure the accuracy of the registration results. This method was also used by ssEMNet to evaluate registration accuracy. For the FIB-mito dataset, this study only uses the NCC of sections to assess the registration results because unlike CREMI dataset, the dataset lacks dense labels of structures.

## 3 Results

This section evaluates the efficacy of the proposed pairwise registration and structural regression methods, as well as the entire framework including the long-serial splitting strategy. The optical flow method used in this study is an improved version of ARFlow. Thus, the original pre-trained ARFlow model (Liu *et al.* 2020) and the ARFlow_ft model fine-tuned on the zebrafish dataset (as described in Section 2.4) are used as the baselines for this experiment. All models are trained with Intel(R) Xeon(R) Gold 6142 CPU and one NVIDIA Tesla V100 GPU.

### 3.1 Pairwise registration

Pairwise registration is the basis of serial registration. It is desirable for pairwise registration methods to prioritize the location changes of biological tissues over morphological changes. In this experiment, for each randomly deformed section$\;S_{\mathrm{moving}}$, this article uses the previous undeformed section as the reference section $S_{\mathrm{reference}}$. More specifically, the pairwise registration estimates the deformation field of the two sections, and then the field is used to warp $S_{\mathrm{moving}}$.

**Figure 5.**
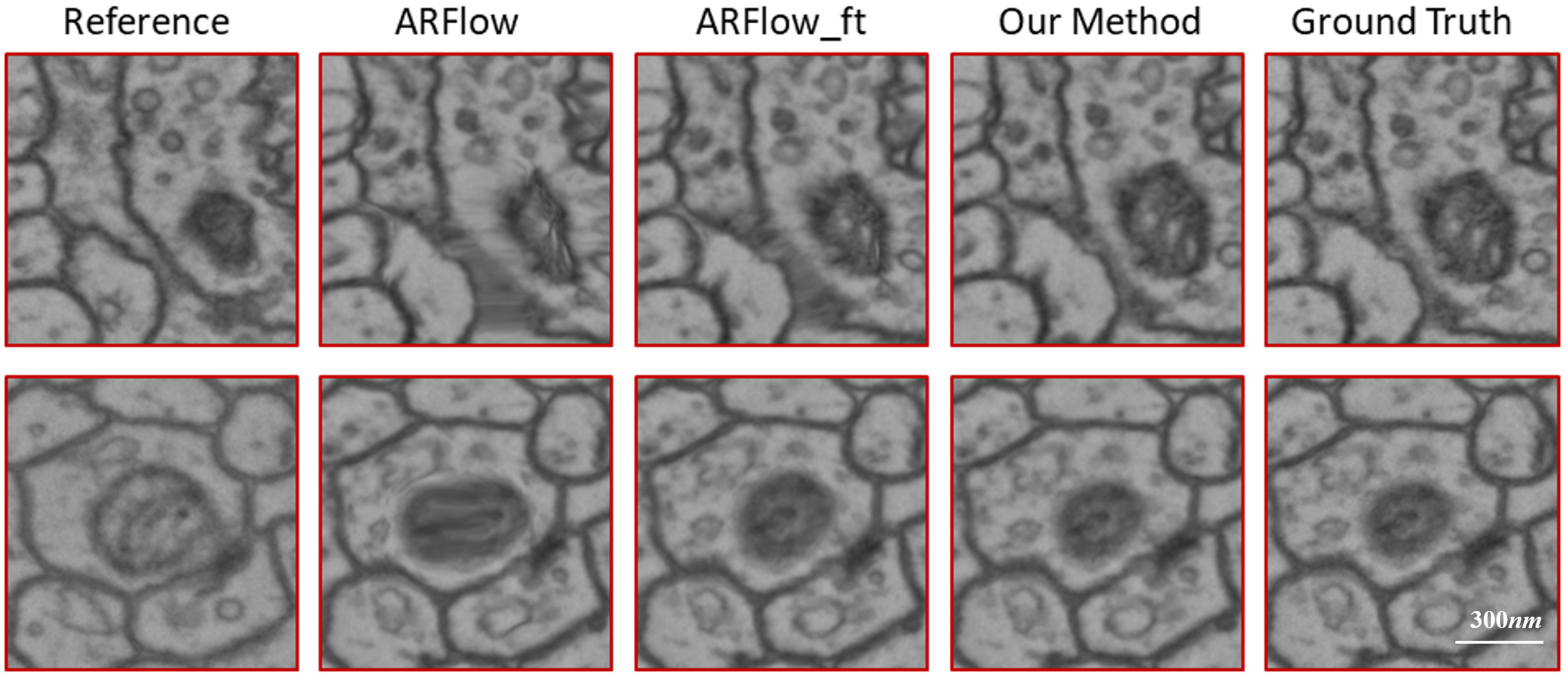
Impact of improved registration method on the morphological changes of two different neurites. The first column shows the patch of reference section, while the middle three columns show the registration results obtained by different methods. The last column shows the ground truth.

Figure 5 shows how our method can improve registration results. In the first row of the figure, mitochondria show a magnified morphological change between the reference section and the section to be registered. In contrast, mitochondria in the second row show a smaller morphological change. After registration, the baseline method tends to distort this natural deformation, while our method retains the morphological change.

This study uses the NCC and Dice coefficient to evaluate the registration results. The NCC measures the pixel similarity between images. While NCC (Reference) indicates the similarity between the warped result and the reference section, it cannot directly reflect the accuracy of the registration. In comparison, NCC (GT) represents the similarity between the result and the ground truth. The Dice coefficient reflects the degree of overlap between the registered neurites and this study uses the NCC and Dice coefficient to evaluate the registration results. The NCC measures the pixel similarity between images. While NCC (Reference) indicates the similarity between the warped result and the reference section, it cannot directly reflect the accuracy of the registration.

In comparison, NCC (GT) represents the similarity between the result and the ground truth. The Dice coefficient reflects the degree of overlap between the registered neurites and the ground truth neurites. To demonstrate the effectiveness of the proposed method, the method is compared with baselines, Elastic (Saalfeld *et al.* 2012), SIFTFlow (Liu *et al.* 2011), FlowFormer (Huang *et al.* 2022), CRAFT (Sui *et al.* 2022), and SEAMLeSS (Mitchell *et al.* 2019). As shown in Table 1, the proposed method exhibits the highest NCC (GT) value and has the highest Dice coefficient.

**Table 1.** Quantitative comparison of pairwise registration.^a^

Method	CREMI				FIB-mito		
	NCC (Reference)	NCC (GT)	Dice coefficient (GT)	Time (s)	NCC (Reference)	NCC (GT)	Time (s)
Elastic (Saalfeld *et al.* 2012)	0.255±0.072	0.300±0.106	0.786±0.097	5.12±0.72	0.273±0.072	0.331±0.093	5.03±0.64
SIFTFlow (Liu *et al.* 2011)	0.827±0.015	0.682±0.086	0.921±0.017	47.57±0.80	0.723±0.027	0.578±0.062	47.62±0.71
FlowFormer (Huang *et al.* 2022)	0.614±0.036	0.692±0.093	0.935±0.018	0.39±0.02	0.517±0.194	0.533±0.192	0.39±0.01
CRAFT (Sui *et al.* 2022)	0.308±0.053	0.405±0.086	0.856±0.025	0.13±0.03	0.320±0.052	0.431±0.068	0.12±0.02
SEAMLeSS (Mitchell *et al.* 2019)	0.615±0.050	0.575±0.079	0.888±0.24	10.53±1.53	0.539±0.053	0.514±0.067	10.42±1.52
ARFlow (Liu *et al.* 2020)	0.681±0.047	0.634±0.091	0.921±0.019	0.09±0.02	0.585±0056	0.498±0.077	0.08±0.16
ARFlow_ft	0.694±0.037	0.689±0.093	0.931±0.018	0.09±0.03	0.599±0.047	0.551±0.080	0.08±00.01
Our Flow	0.575±0.040	**0.705±0.094**	**0.939±0.018**	0.09±0.03	0.484±0.056	**0.585±0.085**	0.09±0.02

a NCC (Reference) is not a reliable indicator of registration performance, a high value only means that it is more similar to the reference section. Higher values of NCC (GT) and Dice (GT) indicate better registration results.

The highest performances are shown in bold.

In addition, an experiment is also conducted to compare the improved method with the baseline method. As shown in Table 1, when comparing the improved method with the baseline methods (ARFlow and ARFlow_ft), it can be seen that ARFlow_ft has the highest NCC (Reference), while the improved method has the highest NCC (GT). This suggests that the registration result of ARFlow_ft is most similar to the reference section, while the registration result of the improved method is closer to the ground truth. This indicates that the improved network is able to better preserve the natural morphology of the neurites.

### 3.2 Serial registration

This study evaluates the effectiveness of the proposed method for serial registration using the first 32 layers of each dataset. The efficacy of the proposed method is quantitatively analyzed and compared with baselines, Elastic (Saalfeld *et al.* 2012), SIFTFlow (Liu *et al.* 2011), FlowFormer (Huang *et al.* 2022), CRAFT (Sui *et al.* 2022), and SEAMLeSS (Mitchell *et al.* 2019). These comparison methods use the serial constraint method mentioned in the original paper, and do not use serial constraint if not mentioned. The baseline method (ARFlow and ARFlow_ft) in this article utilizes the structural regression proposed in this study as the serial constraint method, as it can be seen in Fig. 7 that the serial registration results of the baseline method can be improved by structural regression. The serial registration results are presented in Table 2, where the proposed method achieves the highest registration accuracy across different datasets.

**Table 2.** Quantitative results of serial registration.

Method	CREMI			FIB-mito	
	NCC	Dice coefficient	Time (s)	NCC	Time (s)
Elastic	0.246±0.154	0.782±0.059	436.48	0.233±0.173	437.10
SIFTFlow	0.192±0.177	0.707±0.083	1176.64	0.282±0.166	1172.48
FlowFormer	0.103±0.218	0.410±0.341	12.00	0.113±0.218	12.41
CRAFT	0.443±0.134	0.869±0.033	5.46	0.454±0.135	4.59
SEAMLeSS	0.436±0.132	0.864±0.034	831.86	0.456±0.126	830.33
ARFlow	0.161±0.204	0.521±0.198	15.41	0.164±0.186	13.55
ARFlow_ft	0.451±0.160	0.844±0.046	12.67	0.384±0.135	9.21
Ours	**0.544±0.144**	**0.880±0.033**	12.59	**0.517±0.124**	14.41

The highest performances are shown in bold.

**Figure 6.**
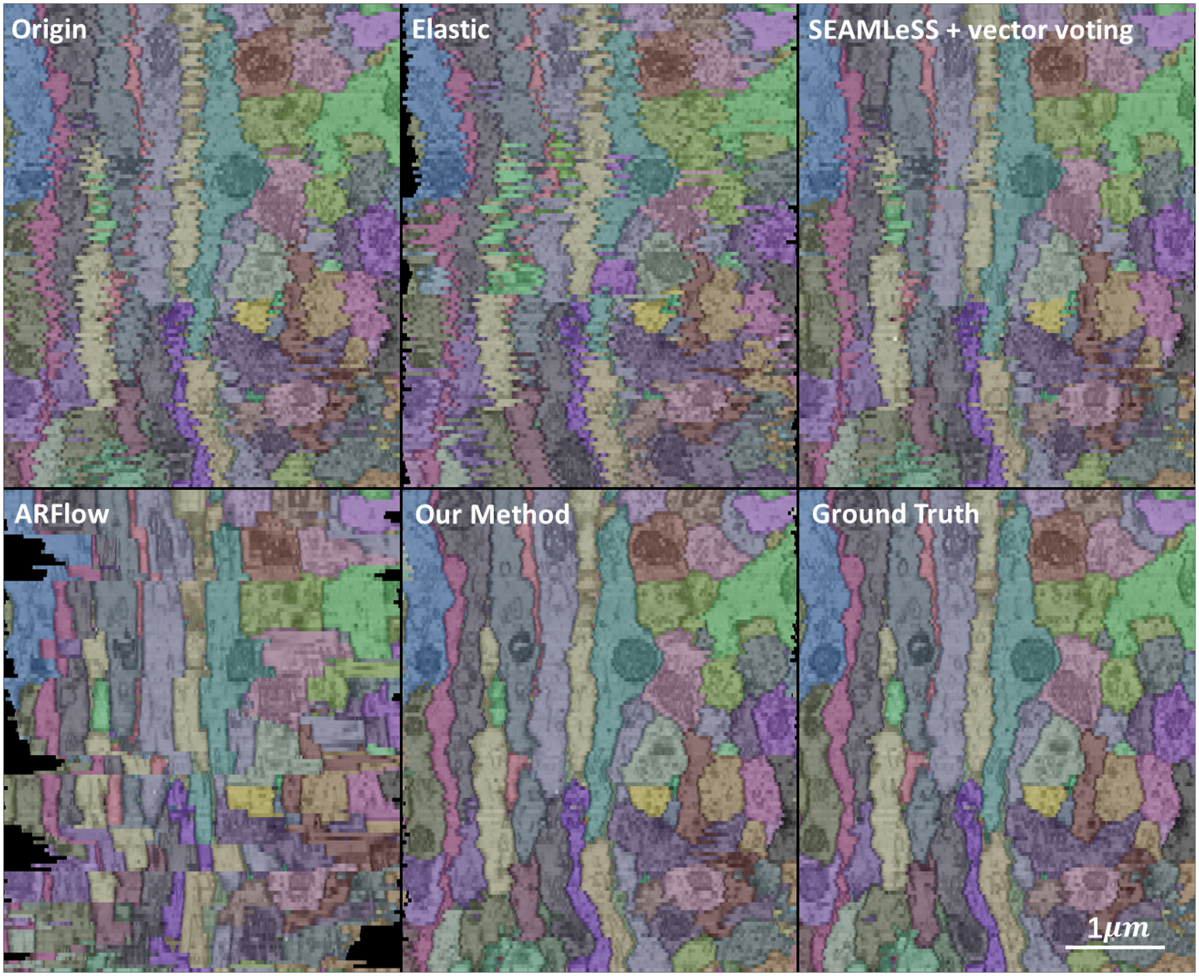
Longitudinal view of long-serial registration results.

The proposed method is specifically designed for short serial section registration, but it can still be used to register long-serial sections by dividing them into multiple short serials. To better demonstrate the performance of serial registration, this study displays a qualitative longitudinal view of long-serial registration results. In this experiment, the CREMI dataset is divided into five short series, each containing 25 sections, using the long-serial division and benchmark sections registration strategy mentioned in Section 2.3. Different serial registration methods are performed on each short series, and the results of the short serial registration are then stacked to construct a long-serial registration. The performance of the proposed method is compared with other registration methods, as shown in Fig. 6, and our method achieves the best longitudinal continuity in the long-serial sections and produces results closest to the ground truth.

### 3.3 Ablations

A set of ablation experiments are conducted in this study to illustrate the impact of each module on serial registration. These experiments are carried out on the first 32 layers of each dataset mentioned in Section 3.2. Table 3 shows the results of these experiments, where each section of the table corresponds to a particular module of the proposed method that was individually tested. The final version of the proposed method employs the versions with underlines. In the following paragraphs, each experiment will be described in detail.

**Table 3.** Ablation experiments.^a^

Experiment	Modules	CREMI		FIB-mito
		NCC	Dice	NCC
Finetune	No	0.161±0.204	0.521±0.198	0.164±0.186
	Yes	0.451±0.160	0.844±0.046	0.384±0.135
Similarity measurement	L1+ssim+ternary	0.451±0.160	0.844±0.046	0.384±0.135
	L2 of UTR descriptors	0.544±0.144	0.880±0.033	0.517±0.124
Serial constraint	No	0.134±0.205	0.591±0.181	0.286±0.180
	Structural regression	0.544±0.144	0.880±0.033	0.517±0.124

a Settings used in our final model are underlined. See Section 3.3 for details.

*Finetune*: Although each optical flow method claims to have good generalization performance, finetuning existing pre-trained models for biological images is significant. The results show that the performance of the model is greatly improved after finetuning on the zebrafish dataset.

*Similarity measure*: This article replaces the pixel-level similarity measure used in the original ARFlow with the L2 distance of UTR descriptors. Training the network using the original similarity measure makes the network susceptible to differences in neural structure and morphology. The results indicate that using the L2 distance of UTR descriptors to measure slice similarity improves serial registration.

*Serial constraint*: The proposed method uses structural regression for serial constraint. Table 3 demonstrates the impact of structural regression on serial registration, with significant improvement shown.

**Figure 7.**
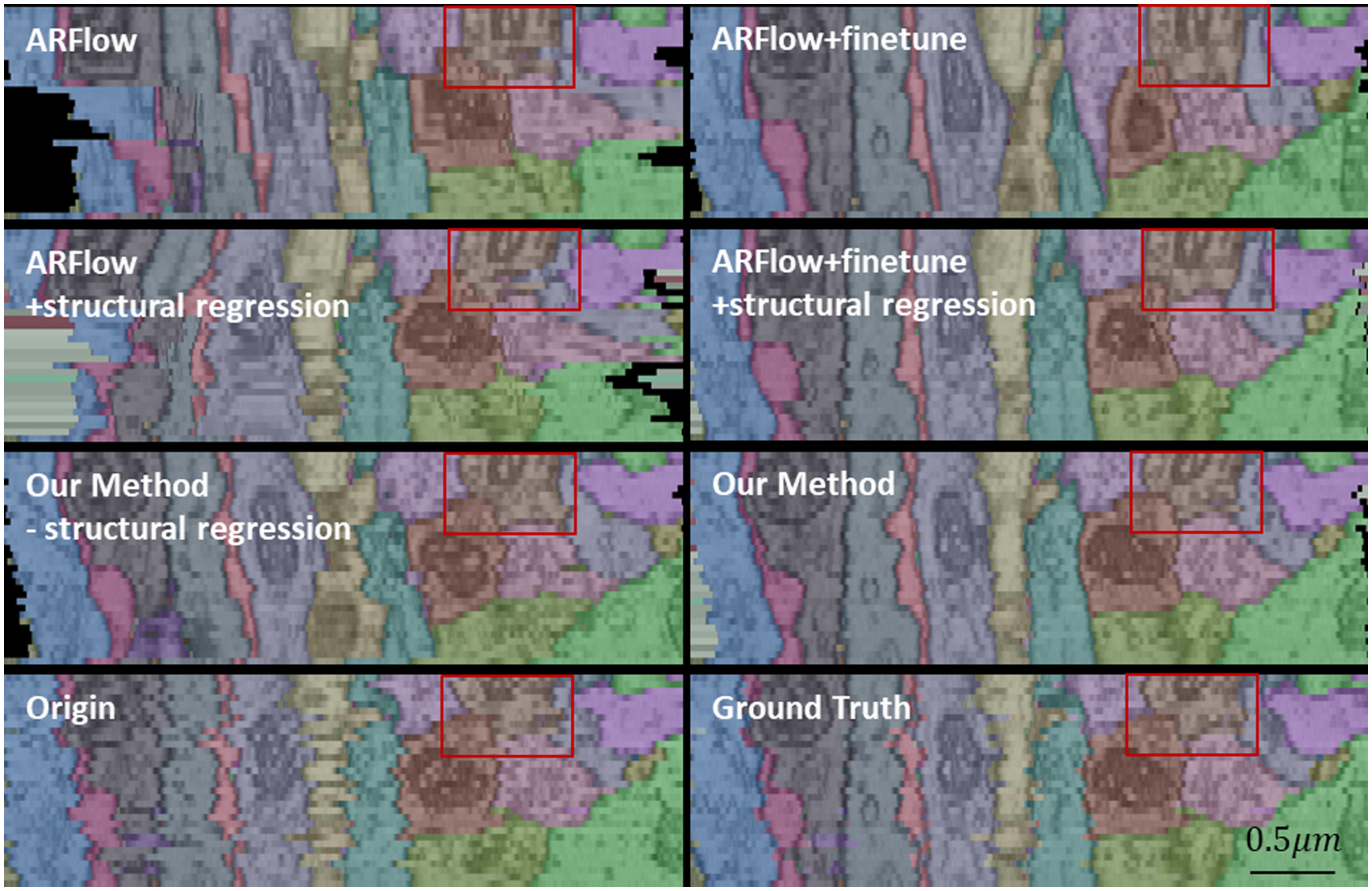
Comparison of the longitudinal view of registration results with different modules in our method (different colors represent different neurites).

Figure 7 illustrates the contribution of each module in the proposed method through qualitative results. This figure presents a longitudinal view of the serial registration results for the first 32 sections of the CREMI dataset. The morphological differences of the neurites in the red box demonstrate that fine-tuned optical flow, UTR descriptors, and structural regression all help to make the reconstructed neurite structure more similar to the ground truth. Moreover, our result shows improved continuity of the cell membrane when compared to the manual registration result.

## 4 Discussion

This study presents a new method for improving the accuracy of serial registration in connectomics research using ssEM. Serial section registration is a crucial step in reconstructing biological tissue volumes, as it tries to prevent artificial deformations and restore the correct neurite structure. However, traditional approaches of serial section registration, such as template matching and optical flow methods, have faced significant challenges, including the risk of overregistration due to optimization based on pixel-level similarity and the generation of serial registration cumulative errors.

This study presents a novel approach to addressing the challenges previously mentioned. The proposed method leverages a stronger representation of features and an unsupervised optical flow network to register pairs of sections. By relying on feature similarity rather than pixel similarity, the network is able to eliminate nonlinear deformations and accurately register the sections while preserving the natural morphological variations of neurites. It also has preferable resistance to scenes, such as contrast blur, lens distortion, and section contamination, it is because UTR (the descriptor used in the article) can suppress the influence of these factors during the training process to some extent. As demonstrated in Fig. 5, the method is able to accurately detect changes in mitochondrial size, which is a challenge for the baseline method. This suggests that the proposed method can correct positional deviations and maintain the normal structure of organelles. Moreover, an optical flow network is used to estimate and compensate for cumulative registration errors, allowing for more accurate recovery of the spatial structure of biological tissues. As shown in Fig. 7, the structural regression technique effectively compensates for cumulative error and corrects deviated neurites to their normal positions.

However, it should be noted that the error accumulation assumption of the structural regression method is based on short serial sections, making it suitable only for short serial section registration. For long-serial section registration, a feasible strategy is to divide the long series into multiple short series for registration. This allows for the reconstruction of the spatial continuity of the entire block. While the short serial splitting strategy proposed in this article is simple, it may be worth considering other feasible schemes. Further study is needed to understand how to effectively connect the relationships between the short serials. In addition, this method cannot solve the registration accuracy problem caused by section fold and crack, which is also a problem that we need to further solve in the future.

The experimental results show that the enhancements proposed in this study effectively improve the spatial continuity of serial sections, resulting in improved registration and accurate recovery of the spatial structure of biological tissues. This method represents a significant advancement in the field of serial section registration, as it addresses the challenges faced by current approaches and has the potential to greatly improve the accuracy of volume reconstruction in the connectomics research.

## References

[btad436-B1] Anderson JR , Jones BW, Yang J-H et al A computational framework for ultrastructural mapping of neural circuitry. PLoS Biol 2009;7:e1000074. 19855814 10.1371/journal.pbio.1000074 PMC2661966

[btad436-B2] Balakrishnan G , Zhao A, Sabuncu MR et al VoxelMorph: a learning framework for deformable medical image registration. IEEE Trans Med Imaging 2019;38:1788–800. 10.1109/TMI.2019.2897538 30716034

[btad436-B3] Bock DD , Lee W-CA, Kerlin AM et al Network anatomy and in vivo physiology of visual cortical neurons. Nature 2011;471:177–82. 21390124 10.1038/nature09802 PMC3095821

[btad436-B4] Bookstein FL. Principal warps: thin-plate splines and the decomposition of deformations. IEEE Trans Pattern Anal Machine Intell 1989;11:567–85.

[btad436-B5] Briggman KL , Bock DD. Volume electron microscopy for neuronal circuit reconstruction. Curr Opin Neurobiol 2012;22:154–61. 22119321 10.1016/j.conb.2011.10.022

[btad436-B6] Cardona A et al An integrated micro-and macroarchitectural analysis of the drosophila brain by computer-assisted serial section electron microscopy. PLoS Biol 2010;8:e1000502. 20957184 10.1371/journal.pbio.1000502 PMC2950124

[btad436-B3855720] Denk W, , Horstmann H. Serial block-face scanning electron microscopy to reconstruct three-dimensional tissue nanostructure. *PLoS Biol* 2004;2:e329. 10.1371/journal.pbio.0020329. 15514700 PMC524270

[btad436-B7] Gardella D , Hatton WJ, Rind HB et al Differential tissue shrinkage and compression in the z-axis: implications for optical disector counting in vibratome-, plastic- and cryosections. J Neurosci Methods 2003;124:45–59. 12648764 10.1016/s0165-0270(02)00363-1

[btad436-B8] Hildebrand DGC , Cicconet M, Torres RM et al Whole-brain serial-section electron microscopy in larval zebrafish. Nature 2017;545:345–9. 28489821 10.1038/nature22356 PMC5594570

[btad436-B9] Huang Z et al FlowFormer: A Transformer Architecture for Optical Flow. Cham, Switzerland: Springer Nature, 2022, 668–85.

[btad436-B0097018] Knott G, , Marchman H, , Wall D et al Serial section scanning electron microscopy of adult brain tissue using focused ion beam milling. *J Neurosci* 2008;28:2959–64. 10.1523/JNEUROSCI.3189-07.2008. 18353998 PMC6670719

[btad436-B10] Liu C , Yuen J, Torralba A. SIFT flow: dense correspondence across scenes and its applications. IEEE Trans Pattern Anal Mach Intell 2011;33:978–94. 20714019 10.1109/TPAMI.2010.147

[btad436-B11] Liu L , Zhang J, He R, et al Learning by analogy: reliable supervision from transformations for unsupervised optical flow estimation. In: *Proceedings of the IEEE/CVF Conference on Computer Vision and Pattern Recognition, Seattle, WA, USA*. 2020, 6489–98.

[btad436-B12] Lucchi A , Li Y, Fua P. Learning for structured prediction using approximate subgradient descent with working sets. In: *Proceedings of the IEEE Conference on Computer Vision and Pattern Recognition, Portland, OR, USA, June 23-28, 2013*. 2013, 1987–94.

[btad436-B13] Lv Y , Chen X, Shu C, et al Robust global optimized affine registration method for microscopic images of biological tissue. In: *ICASSP 2020—2020 IEEE International Conference on Acoustics, Speech and Signal Processing (ICASSP), Barcelona, Spain, May 4-8, 2020*. 2020, 1070–4.

[btad436-B14] Macrina T , Lee K, Lu R, et al Petascale neural circuit reconstruction: automated methods. bioRxiv, 2021, 2021.2008.2004.455162, preprint: not peer reviewed.

[btad436-B15] Mishchuk A et al Working hard to know your neighbor's margins: local descriptor learning loss. In: *Advances in Neural Information Processing Systems 30 (Nips 2017), Long Beach, CA, USA*, Vol. 30. 2017.

[btad436-B16] Mitchell E et al Siamese encoding and alignment by multiscale learning with self-supervision. arXiv, arXiv:1904.02643, 2019, preprint: not peer reviewed.

[btad436-B17] Popovych S , Bae JA, Seung HS. Caesar: segment-wise alignment method for solving discontinuous deformations. In: *2020 IEEE 17th International Symposium on Biomedical Imaging (ISBI)*. IEEE, Iowa City, IA, USA, April 3-7, 2020, 2020, 1214–8.

[btad436-B18] Saalfeld S , Fetter R, Cardona A et al Elastic volume reconstruction from series of ultra-thin microscopy sections. Nat Methods 2012;9:717–20. 22688414 10.1038/nmeth.2072

[btad436-B19] Shapson-Coe A , Januszewski M, Berger D R, et al A Connectomic Study of a Petascale Fragment of Human Cerebral Cortex. Cold Spring Harbor Laboratory, 2021.

[btad436-B20] Sui X et al CRAFT: cross-attentional flow transformer for robust optical flow. In: *Proceedings of the IEEE/CVF Conference on Computer Vision and Pattern Recognition, New Orleans, LA, USA, June 18-24, 2022*. 2022, 17602–11.

[btad436-B21] Sun D et al PWC-Net: CNNs for optical flow using pyramid, warping, and cost volume. In: *2018 IEEE/CVF Conference on Computer Vision and Pattern Recognition, Salt Lake City, UT, USA, June 18-22, 2018*. 2018, 8934–43.

[btad436-B22] Tasdizen T , Koshevoy P, Grimm BC et al Automatic mosaicking and volume assembly for high-throughput serial-section transmission electron microscopy. J Neurosci Methods 2010;193:132–44. 20713087 10.1016/j.jneumeth.2010.08.001 PMC2952705

[btad436-B23] Tian YR , Fan B, Wu FC. L2-Net: deep learning of discriminative patch descriptor in Euclidean space. *30th IEEE Conference on Computer Vision and Pattern Recognition (CVPR 2017), Honolulu, HI, USA, July 21-26, 2017*. 6128–36. 2017.

[btad436-B24] Xin T et al UTR: Unsupervised Learning of Thickness-Insensitive Representations for electron microscope image. In: *2021 IEEE International Conference on Image Processing (ICIP)*. IEEE, Anchorage, AK, USA, September 19-22, 2021, 2021.

[btad436-B25] Xin T , Shen L, Li L et al Expected affine: a registration method for damaged section in serial sections electron microscopy. Front Neuroinform 2022;16:944050. 36120082 10.3389/fninf.2022.944050 PMC9478550

[btad436-B26] Yoo I et al ssEMnet: serial-Section electron microscopy image registration using a spatial transformer network with learned features. In: Cardoso MJ (ed.), Deep Learning in Medical Image Analysis and Multimodal Learning for Clinical Decision Support. Cham: Springer International Publishing, 2017, 249–57.

[btad436-B27] Zheng Z , Lauritzen JS, Perlman E et al A complete electron microscopy volume of the brain of adult Drosophila melanogaster. Cell 2018;174:730–43.e22. 30033368 10.1016/j.cell.2018.06.019 PMC6063995

